# *Trichoderma harzianum* Strain T22 Modulates Direct Defense of Tomato Plants in Response to *Nezara viridula* Feeding Activity

**DOI:** 10.1007/s10886-021-01260-3

**Published:** 2021-03-13

**Authors:** Tuğcan Alınç, Antonino Cusumano, Ezio Peri, Livio Torta, Stefano Colazza

**Affiliations:** Department of Agricultural, Food and Forest Sciences, University of Palermo, Viale delle Scienze, Bld. 5, 90128 Palermo, Italy

**Keywords:** Stink bugs, Beneficial soil microbes, *Solanum lycopersicum*, Jasmonic acid signaling pathway, Pentatomidae

## Abstract

Plant growth-promoting fungi belonging to genus *Trichoderma* are known to help plants when dealing with biotic stressors by enhancing plant defenses. While beneficial effects of *Trichoderma* spp. against plant pathogens have long been documented, fewer studies have investigated their effect on insect pests. Here, we studied the impact of *Trichoderma* root colonization on the plant defense responses against stink bug feeding attack. For this purpose, a model system consisting of tomato plant, *Solanum lycopersicum* cv Dwarf San Marzano, *Trichoderma harzianum* strain T22 and the southern green stink bug, *Nezara viridula*, was used. We firstly determined stink bug performance in terms of relative growth rate and survival on tomato plants inoculated by *T. harzianum* T22. Then, we evaluated relative expression of plant defense-related genes on inoculated plants induced by stink bug feeding. We found evidence that *T. harzianum* T22 affects tomato defense responses against *N. viridula* nymphs leading to reduction of growth rate. Our results also showed that *T. harzianum* T22 enhances plant direct defenses by an early increase of transcript levels of jasmonic acid marker genes. Yet this effect was time-dependent and only detected 8 h after herbivore induction. Taken together, our findings provide better understanding on the mechanisms underlying tomato induced resistance against herbivorous stink bugs.

**Supplementary Information:**

The online version contains supplementary material available at 10.1007/s10886-021-01260-3.

## Introduction

Plants can mount direct and indirect defense strategies by activating their immune system after herbivorous insect attack (Walling 2000). Direct defenses negatively affect the performance of herbivores feeding on plants via physical (e.g. trichomes, thorns and waxes) and/or chemical mechanisms (e.g. toxins and deterrents) (Howe and Schaller 2008). Indirect defenses enhance the recruitment of natural enemies of the attacking herbivore via emission of herbivore-induced plant volatiles (D’Alessandro and Turlings 2006; Kessler and Baldwin 2001; Rasmann et al. 2005). It is increasingly recognized that plants are not alone when interacting with herbivorous insects as they have established, over evolutionary time, associations with microbial symbionts that could influence plant defenses against herbivory (Pineda et al. 2010; Sugio et al. 2015). In fact, several studies have indicated that plant defenses can be promoted by beneficial soil microbes, such as plant growth-promoting rhizobacteria (Dicke and Hilker 2003; Kessler and Baldwin 2001), mycorrhizal fungi (Pozo and Azcón-Aguilar 2007), endophytic fungi (Stein et al. 2008) and plant growth-promoting fungi (Harman et al. 2004; Segarra et al. 2009).

Beneficial soil microbes have been used in agriculture due to their positive effects on plant survival, growth and yield through direct or plant-mediated mechanisms (Bender et al. 2016; Rodriguez and Sanders 2015). However, these microbes do not only promote nutrient acquisition and improve tolerance to abiotic stresses, but they also lead to negative effects on aboveground and belowground biotic stressors (Guerrieri and Digilio 2008; Pieterse et al. 2014). Among beneficial soil microbes, plant growth-promoting fungi (PGPFs) belonging to genus *Trichoderma* are well known as effective widespread biological control agents against plant pathogens (Harman et al. 2004; Woo et al. 2014). Whereas the defensive effect of *Trichoderma* spp. on plant pathogens is well-document (Hanson and Howell 2004; Vinale et al. 2012), recent studies have also revealed their role in mediating plant defenses against insect herbivores, in particular against piercing-sucking pests. For example, phloem-feeding aphid survival was significantly reduced by tomato root colonization by *Trichoderma atroviride* strain P1 (Coppola et al. 2019a). Similarly, the performance of the cell-content feeder *Thrips tabaci* Lindeman is negatively affected by onion plants colonized by *Trichoderma* spp. (Muvea et al. 2014). The population of the phloem-feeding insect *Trialeurodes vaporariorum* (Westwood) was found to decline after inoculation with either *T. atroviride* MT-20, *T. atroviride* S-2 and *Fusarium oxysporum* (Fo162) (Menjivar et al. 2012).

Beneficial soil microbes affect plant defenses via alteration of signaling pathways that enhance plant gene expression and metabolism in order to inhibit development of biotic stressors (Pineda et al. 2010; van de Mortel et al. 2012; Verhagen et al. 2004). In the regulation of plant defenses, phytohormones such as jasmonic acid (JA), salicylic acid (SA) and ethylene (ET) play a key role against pathogens and herbivores (Pieterse and Dicke 2007). JA is acquired from linolenic acid through octadecanoid pathway and triggers direct defenses to wounding, necrotrophic pathogens and herbivores, commonly chewing insects (Pieterse et al. 2012; Walling 2000; Zhang et al. 2017). Chewing of plant parts by insects causes the deoxygenation of linolenic acid and activates an octadecanoid pathway that results in JA biosynthesis eventually leading to production of proteinase inhibitors, polyphenol oxidases, plant-specific toxins (e.g. glucosinolates, alkaloids, terpenoids) and attraction of insect parasitoids (Broadway and Duffey 1986; Walling 2000; War et al. 2012). However, defense-signaling pathways can be tailored depending on insect species adaptation to host plant or feeding mode (Moran and Thompson 2001; Stotz et al. 2000). Thus, while JA signaling pathway is activated in response to mostly chewing herbivores, piercing-sucking insects induce mainly SA-related defenses (Walling 2000). Recent studies have showed that *Trichoderma* spp. can induce systemic resistance by enhancing phytohormone signaling pathways and defense priming in plants (Conrath et al. 2015; Yuan et al. 2019). For example, root colonization by *T. atroviride* P1 induced a plant transcriptome reprogramming in which both SA and JA pathways were up-regulated (Coppola et al. 2019a). Yet another study showed that *T. harzianum* T78 limited the root knot nematode, *Meloidogyne incognita* (Kofoid and White) Chitwood, infection cycle (i.e. galling, fecundity and root invasion) through priming the SA- and the JA-dependent pathways in tomato roots (Martínez-Medina et al. 2017).

Stink bugs (Heteroptera: Pentatomidae) are considered a group of major pests in several crops which cause economically important yield losses worldwide (Conti et al. 2021). To date, limited evidence is available about direct defenses of plants against stink bug damage which suggests the involvement of both JA and SA signaling pathways. For example, both SA and JA signaling pathways in *Arabidopsis thaliana* (L.) plants are activated in response to *Eurydema oleracea* (L.) feeding (Ederli et al. 2020). A combination of oviposition and feeding activities by the brown marmorated stink bug, *Halyomorpha halys* Stål, induces the JA pathway in *Vicia faba* L. plants resulting in the activation of cysteine protein inhibitor genes and *NAI1* (Rondoni et al. 2018).

To the best of our knowledge, the role of beneficial soil microbes in enhancing plant defenses against stink bug feeding has not been investigated. Thus, unravelling the potential role of beneficial soil microbes against such piercing-sucking herbivores needs attention to provide a better insight on multitrophic interactions, which may help to develop new strategies for controlling these important pests. This was the aim of the study in which we investigated: (i) the performance of stink bug nymphs (both in terms of relative growth rate and survival) on tomato plants after root inoculation by beneficial soil microbe; (ii) the underlying molecular mechanism by which beneficial soil microbe can affect pest performances. We explored the above-mentioned objectives using a multitrophic system consisting of the tomato plant, *Solanum lycopersicum* L.*,* the PGPF *T. harzianum* T22 and the piercing-sucking herbivore *Nezara viridula* (L.)*. Trichoderma harzianum* T22 is one of the *Trichoderma* strains that hold potential for sustainable crop production and is available as commercial product (Vitti et al. 2015) and *N. viridula* is a serious insect pest of tomato feeding on the leaves and fruits causing discoloration upon ripening and development of corky area below the fruit surface (Wakil et al. 2017).

## Materials and Methods

### Fungal Cultures, Insects and Plants

*Trichoderma harzianum* T22 was provided by University of Naples Federico II, Naples, Italy. The isolate was routinely grown and sub-cultured on potato dextrose agar (PDA Oxoid) under room conditions. Spores were harvested from PDA plates by flooding with sterile distilled water and adjusted to 10^7^ ml^−1^ spores/ml conidial suspension.

The colony of *N. viridula* was reared in insect cages (47.5 × 47.5 × 47.5 cm) (Bug-Dorm-44,545, MegaView Science Co. Ltd., Taichung, Taiwan) under controlled conditions (24 ± 1 °C; 70 ± 5% RH and 14L:10D photoperiod). Insects were fed with fresh organic vegetables, sunflower and zucchini seeds. Water was provided as soaked cotton wool inside a 12-cm petri dish and paper towels were placed into cages as oviposition substrates. Food was renewed every 2–3 days and newly laid eggs were collected on a daily basis to maintain the colony.

Tomato (*S. lycopersicum*) cv ‘Dwarf San Marzano’ was used for all experiments. The plants were kept in a growth chamber following transplant procedures (see below for details) and watered every other day. The growth chamber was set at 23 ± 2 °C 70 ± 5% RH and 14L:10D photoperiod condition and equipped with the light bulbs placed above the foliage providing a photosynthetic flux density of 600 mol photons m^−2^ s^−1^. Each plant in the experiments was around 20 cm height with 3–4 fully expanded leaves.

### Seed Treatment

Seed treatment of tomato was carried out as described by Coppola et al. (2019b). The surface of seeds was sterilized using 1% (*v*/v) sodium hypochlorite for 5 min and properly rinsed in sterile distilled water. The seeds were coated with a 10^7^ sp. ml^−1^ conidial suspension of *T. harzianum* T22 or with water for control. Following air desiccation for 24 h, dried seeds were placed on water-moistened filter paper in a sterile petri dish kept in the dark at 25 °C. Germinated seedlings were the transferred in sterilized soil filled trays and maintained in a growth chamber at 23 ± 2 °C, 70 ± 5% RH and 14L:10D photoperiod condition. After 3 weeks, tomato seedlings were transplanted into 14-cm-diameter plastic pots.

### Insect Performance Bioassays

Insect performance on tomato plants colonized by *T. harzianum* T22 was investigated by exposing each plant (inoculated or water control) to 3rd instar nymphs of *N. viridula*. Plants with 3–4 fully expanded leaves were enclosed together with 5 insects inside a nylon mesh bag (size = 30 cm × 40 cm; mesh count = 300 mesh/cm^2^). The nymphs were weighed on a Kern ABS-N analytical balance (Kern & Sohn, Germany) prior to bioassays and then allowed to feed on the plants for 1 week under controlled conditions (24 ± 1 °C; 70 ± 5% RH and 14L:10D photoperiod). After 1-week, nymphs were removed and re-weighed. To assess insect performance, we calculated for each plant, nymph mortality (i.e. % dead nymphs in relation to total nymphs) and nymph relative growth rate. To keep into account mortality effects on relative growth rate, we averaged the weight of the nymphs that were initially enclosed in the plant (i.e. initial average weight) as well as the weight of the nymphs that survived at the end of the experiment (i.e. final average weight). Thus, nymph relative grow rate was recorded as: (final average weight - initial average weight)/ initial average weight * 100). For each treatment, 15 replicates were carried out.

### Plant Induction, Isolation of RNA and qPCR

To study tomato plant responses mediated by *T. harzianum* T22 against *N. viridula*, we quantified relative transcript levels of three defense marker genes involved in different signal-transduction pathways. As a marker of JA-signaling pathway we used *ToLOX D* whereas, as a marker of SA-signaling pathway, we used *ToPR-1*. We also investigated transcript levels of *ToPIN2*, a gene coding for a protein inhibitor.

Tomato plants were treated by exposing the youngest fully expanded leaf to a 4–5 d-old female of *N viridula*. Insects were individually confined on the leaf surface using a clip cage (3.8 cm diameter; 1 cm high) with a mesh-covered hole (3 cm diameter) and with the rim covered by a sponge ring to prevent damage to the leaf. The insects were allowed to feed for 8, 24 or 72 h, then the clip cages and the insects were removed, and leaf disks (3.8 cm in diameter) were excised from the treated leaf in order to be stored at −80 °C until gene expression analyses. Tomato plants were either inoculated with *T. harzianum* T22 at seed stage as described above and then induced by stink bug feeding (Treatment “T22Nv”) or were only exposed to stink bug feeding (Treatment “Nv”). As control we used leaf disks collected from plants with empty clip-cages to assess gene expression level in the absence of herbivory (undamaged, non-inoculated plants). Five biological replicates, each consisting of a leaf disk per plant and per treatment, were performed. RNA was isolated using the ISOLATE II Plant RNA kit from Bioline according to the manufacturer’s instructions. Two μg of total RNA was reverse-transcribed into cDNA using Bio-Rad’s iSCRIPT cDNA synthesis kit in a 40 μL reaction volume according to the manufacturer’s instructions. Primers (Table S1) were earlier designed (Coppola et al. 2015; De Palma et al. 2016). iQ SYBRGreen Supermix (Bio-Rad) was used to perform the real time qPCR reactions in duplicate. The following PCR program was used for all PCR reactions: 95 °C for, 3 min followed by 40 cycles of 95 °C for 10 s, annealing temperature of 62 °C for 10 s and 72 °C for 30 s, with data collection at 72 °C. The PCR reactions were followed by a melt curve analysis to check for primer-dimer formation or unspecific PCR products. Relative changes in gene expression were assessed with the 2^- ΔΔCq^ method (Livak and Schmittgen 2001). Delta-delta Cq values were calculated using the quantification cycle (Cq) values of the untreated plants and normalizing using the Cq values of the reference gene Actin.

### Statistical Analysis

Logistic regression with binomial error distribution and log-link function was used to test whether stink bug mortality was affected by the *T. harzianum* T22 inoculation treatment. ANOVA was used to test whether stink bug relative growth rate on tomato plants was affected by fungal inoculation treatment. ANOVA was also used to test if transcript levels of plant genes were significantly affected by the treatments and by different stink bug feeding times (8, 24 and 72 h). Gene transcription data were log-transformed before analyses to meet assumptions of normality and heteroscedasticity and model fit was assessed with residual plots. Post-hoc differences between the treatments were tested using Tukey tests. Data were analyzed with R statistical software (R Development Core Team 2013).

## Results

### Effect of *Trichoderma harzianum* T22 on Insect Performance

**Fig. 1:**
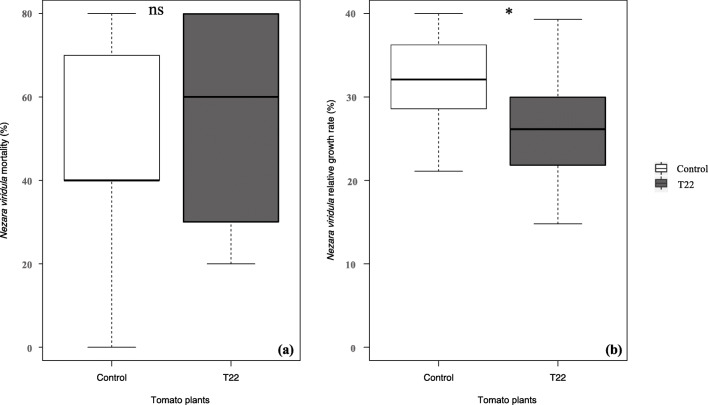
Percentage of mortality (**a**) and relative growth rate (**b**) of *Nezara viridula* 3rd instar nymphs feeding on *Trichoderma harzianum* T22 inoculated (T22) versus non-inoculated (Control) tomato plants. Bold horizontal lines show medians, boxes contain the 25th–50th percentiles, whiskers show the upper and lower quartiles and points show outliers (ANOVA, *P* < 0.05, ns = no significant differences)

Inoculation of tomato plants with *T. harzianum* T22 did not affect the mortality of *N. viridula* 3rd instar nymphs (GLM, χ^2^ = 0.05, df = 1, *P* = 0.827) (Fig. 1a). However, a significant effect of *T. harzianum* T22 was found in terms of relative growth rate (ANOVA, *F* = 5.49, df = 1,28 *P* = 0.026) as the weight of nymphs feeding on plants inoculated with the fungus was lower than nymphs feeding on non-inoculated plants (Fig. 1b).

### Effect of *Trichoderma harzianum* T22 on Gene Transcript Levels

**Fig. 2:**
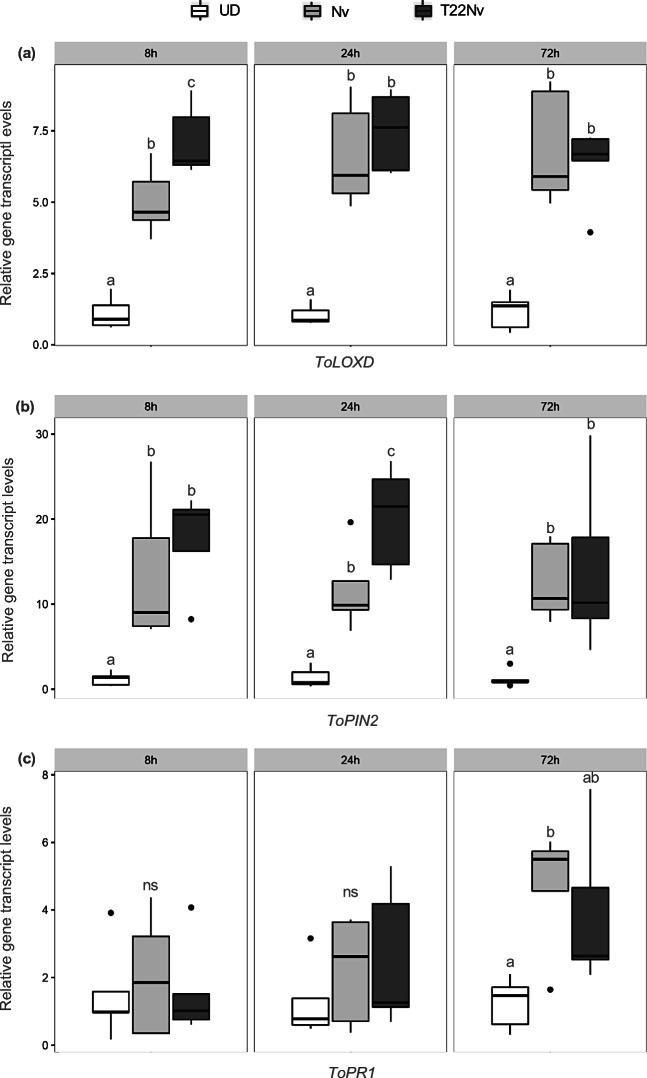
Effects of tomato root colonization by *Trichoderma harzianum* T22 on relative expression of defense marker genes. Expression levels of the JA-marker gene *ToLOX D* (**a**), protein inhibitor gene *ToPIN2* (**b**) and SA-marker gene *ToPR1* (**c**) were analyzed in the leaves of tomato plants at 8 h, 24 h or 72 h. UD = non-inoculated undamaged plants; Nv = non-inoculated plants damaged by feeding of *N. viridula* female; T22Nv = plants inoculated with *T. harzianum* T22 and subsequently damaged by feeding of *N. viridula* female. Bold horizontal lines show medians, boxes contain the 25th–50th percentiles, whiskers show the upper and lower quartiles and points show outliers. Different letters indicate statistically significant differences (ANOVA followed by Tukey post hoc test, *P* < 0.05, ns = no significant differences)

A significant effect of the treatment was found in transcript levels of *ToLOX D* as tomato plants treated with stink bug feeding were upregulated compared with undamaged control plants, regardless of the time point (ANOVA, 8 h: *F* = 43.61, df = 2,12 *P* < 0.001; 24 h: *F* = 33.24, df = 2,12 *P* < 0.001; 72 h *F* 23.00 =, df = 2,12 *P* < 0.001) (Fig. 2a). Yet the beneficial effect of the fungus was found only at 8 h time point with higher *ToLOX D* expression levels (treatment T22Nv compared with treatment Nv) (Fig. 2a). The transcript dynamics of *ToPIN2* were similar to *ToLOX D* as significant differences between plants exposed to stink bug feeding and undamaged plants were found for each time interval (ANOVA, 8 h: *F* = 10.33, df = 2,12 *P* = 0.002; 24 h: *F* = 21.07, df = 2,12 *P* < 0.001; 72 h: *F* = 5.95, df = 2,12 *P* = 0.016). A significant upregulation of *ToPIN2* in the treatment T22Nv compared with Nv was found at 24 h (Fig. 2b). No significant differences in transcript levels of *ToPR1* were found between plants exposed to stink bug feeding and undamaged plants at 8 h and 24 h (ANOVA, 8 h: *F* = 0.15, df = 2,12 *P* = 0.861; 24 h: *F* = 0.76, df = 2,12 *P* = 0.489). A significant treatment effect was found at 72 h (*F* = 5.32, df = 2,12 *P* = 0.022) with non-inoculated plants exposed to *N. viridula* feeding showing higher transcript levels compared to undamaged plants (Fig. 2c). Transcript levels of *ToPR1* were overall similar across time intervals between non-inoculated and inoculated plants induced by stink bug feeding activity.

## Discussion

This study provides the first evidence that root colonization of beneficial soil microbes affects direct defenses of plants in response to stink bug feeding activity. Although *N. viridula* mortality was not enhanced, we observed that the relative growth rate of nymphs was negatively affected when they fed on tomato plants previously inoculated with *T. harzianum* T22. To date, it has been shown that *Trichoderma* spp. colonization affects plant responses against herbivores which induce different feeding damage (Contreras-Cornejo et al. 2018b; Muvea et al. 2014). Plant responses to herbivory have often been linked to feeding patterns with a general dichotomy between piercing-sucking and chewing herbivores (Pieterse et al. 2009; Stam et al. 2014). Yet even within piercing-sucking insects, dissimilar feeding modes occur which could affect the way the plant responds to herbivores (Walling 2000). For example, aphids can use their stylets to access to phloem content, whereas stink bugs can use their stylets to “lacerate and flush” plant tissues (Miles 1972; Velikova et al. 2010). Yet *Trichoderma* spp. fungi have been shown to promote plant defenses against a wide range of insect attackers, regardless of the profound differences in feeding mode and wounding patterns induced by chewing and piercing-sucking insects (Contreras-Cornejo et al. 2018a; Coppola et al. 2019a, 2019b; Menjivar et al. 2012; Muvea et al. 2014).

Few studies have investigated plant molecular responses against stink bug feeding even in the absence of beneficial soil microbes, thus, we first discuss our results showing how non-inoculated tomato plants respond to stink bug feeding and later we focus on how such responses are modulated by *Trichoderma* spp. At the molecular level, we show that tomato plants respond to *N. viridula* feeding by activating the JA-defense signaling pathway detected through an increase in expression levels of *ToLOX D* after 8 h of stink bug feeding. *ToPIN2* was also significantly upregulated already 8 h after herbivore feeding, probably as consequence of the activation of the JA-cascade. Considering that protein inhibitors are known to impair herbivore performance (Coppola et al. 2019a), upregulation of *ToPIN2* may play role in stink bug nymph reduced growth rate. Our results are in agreement with Peiffer and Felton (2014) as they observed significant upregulation in *ToPIN2* expression in plants induced with salivary extract of the *H. halys*. Another study has also shown similar JA-mediated responses (i.e. activation of cysteine protein inhibitor gene and *NAI1*) in broad bean plants induced by feeding and oviposition activity by *H. halys* (Rondoni et al. 2018). Although gene expression patterns of tomato plants exposed to *N. viridula* feeding mainly induce activation of JA-induced defenses, we have evidence that SA-signaling pathway is also involved. In fact, our results demonstrate a significant increase of transcript levels of *ToPR1* after 72 h of *N. viridula* induction. Involvement of SA-defense pathway is also documented for the stink bug *E. oleracea* feeding on *A. thaliana* plants (Ederli et al. 2020), although this study differed with ours in the timing of plant responses to stink bug feeding, since early upregulation of *PR1* in *A. thaliana* plants occurred after 6 h of feeding. Interestingly, the same work found that transcript levels of the JA-dependent gene *AtPDF1.2* were induced later than *AtPR1*: taken together these studies provide evidence that both JA- and SA-defenses are involved in plant responses to stink bug feeding but there seems to be a specificity in terms of temporal patterns of molecular defenses (Ederli et al. 2020).

Concerning the role played by *T. harzianum* T22 at the plant-insect interface, our study indicated that root inoculation of tomato plants with *T. harzianum* T22 affected plant molecular responses to stink bug herbivory. Specifically, we found that inoculated plants significantly increase JA-defense signaling pathway as detected through an early increase (8 h) in *ToLOX D* and in *ToPIN2* (24 h) transcript levels compared with non-inoculated plants. Nonetheless, we did not find any evidence showing that *T. harzianum* T22 boosts SA-defense signaling pathway. In fact, expression levels of *ToPR1* were similar between inoculated and control plants, regardless of the time intervals investigated.

The early activation of JA-defense signaling pathway in plants inoculated with *T. harzianum* T22 indicates that these plants respond faster and more effectively to stink bug feeding. Thus, it is possible to hypothesize that the mechanisms by which *T. harzianum* T22 enhances resistance of tomato plants to *N. viridula* feeding could involve the so-called ‘defense priming’. Priming is a phenomenon which sets the plants in ‘alert’ status ensuring faster and/or stronger defensive responses when attacks by biotic stressors occur (Conrath et al. 2015; Lorito et al. 2010; Martínez-Medina et al. 2017; Van Der Ent et al. 2009). To date, evidence showing that *Trichoderma* spp. prime plant defenses against biotic stressors is largely available for pathogens (Brotman et al. 2013; Gallou et al. 2009; Perazzolli et al. 2011; Yedidia et al. 2003). As in our study, a priming effect due to enhanced JA-defenses was induced by *T. harzianum* T78 in tomato plants challenged by the necrotrophic leaf pathogen *Botrytis cinerea* (Martínez-Medina et al. 2013). Furthermore, induction of a JA-related priming state by *T. harzianum* T22 in tomato plants has been observed against the aphid *Macrosiphum euphorbiae* (Thomas) (Coppola et al. 2019b).

To conclude, this study is the first piece of evidence showing that the strength of plant responses to stink bug feeding is positively affected by root inoculation with PGPFs as *Trichoderma*. Furthermore, we shed new light on the molecular mechanisms underlying the *Trichoderma*-induced resistance in tomato in response to feeding by *N. viridula*. Taken together, these results suggest that the use of beneficial soil microbes to enhance plant defenses in a crop protection perspective appears a promising strategy to control an important group of pests such as herbivorous stink bugs in order to reduce pesticide application. Further works need to be carried out to establish whether beneficial soil microbes affect the indirect plant defenses against stink bugs and chemical communication in multitrophic interactions.

## Supplementary Information

ESM 1 (PDF 37.4 kb)

## Data Availability

The data sets generated during and/or analysed during the current study are available from the corresponding author. Raw data will archived in the Dryad Digital Repository.
