# Supplementary material for: Trichoderma harzianum Strain T22 Modulates Direct Defense of Tomato Plants in Response to Nezara viridula Feeding Activity
Source: J Chem Ecol. 2021 Mar 13;47(4):455–62. doi: 10.1007/s10886-021-01260-3 (PMC8116274; doi:10.1007/s10886-021-01260-3)
Supplement: Supplementary file 1 — (PDF 37.4 kb) [file 10886_2021_1260_MOESM1_ESM.pdf]

Supplementary Table S1. Specific primers for quantitative PCR of plant-defense related genes

Oligoname	Sequence	Name/Gene symbol	Primer from
LoxD Fw	TTCATGGCCGTGGTTGACA	lipoxigenase D (LOX D)	Coppola et at 2015
LoxD Rv	AACAATCTCTGCATCTCCGG		
PIN II Fw	CCAAAAAGGCCAAATGCTTG	Proteinase inhibitor II (PIN II)	Coppola et at 2015
PIN II Rv	TGTGCAACACGTGGTACATCC		
PR1 Fw	ATGCAACACTCTGGTGGACCTT	PR1	Coppola et at 2015
PR1 Rv	CCATTGCTTCTCATCAACCCA		
Actin-fw	CACCACTGCTGAACGGGAA	Actin	De Palma et al 2016
Actin-rev	GGAGCTGCTCCTGGCAGTTT		
